# Melanoma treatment via non-specific adhesion of cancer cells using charged nano-clays in pre-clinical studies

**DOI:** 10.1038/s41598-021-82441-8

**Published:** 2021-02-02

**Authors:** Sahel N. Abduljauwad, Habib-ur-Rehman Ahmed, Vincent T. Moy

**Affiliations:** 1 Civil and Environmental Engineering Department, King Fahd University of Petroleum and Minerals (KFUPM), Dhahran, Saudi Arabia; 2 Department of Physiology and Biophysics, University of Miami, Coral Gables, FL USA

**Keywords:** Cancer therapy, Nanoscale materials

## Abstract

The incidence of malignant melanoma has rapidly increased in the last two decades. There are many challenges associated with the current conventional therapies, including tumour size and location, the specificity of treatments, tumour resistance, non-mutually exclusive mutations, drug resistance, and many adverse side effects. Due to conventional therapies having several limitations, we have explored an alternative therapy such as nano-clays; nano-sized natural materials originating from clay fraction of the soil. Recently, clay nanoparticles have increasingly been used as a drug carrier for cancer treatment due to their high absorption, ability to engulf microbes, and low toxicity. In this study, we evaluated the effects of a nano-clays mix on melanoma cell proliferation and cell viability in vitro and melanoma growth in vivo xenograft animal model. The in vitro study revealed that nano-clay treatments significantly reduced melanoma cell proliferation and cell viability in a dosage-dependent manner. The in vivo tumour xenograft model demonstrated that nano-clay mix treatment led to significantly reduced tumour size and weight, decreased tumour cell mitosis, and induced tumour necrosis. These processes owe to the most probable changes in the membrane potential of the cancer cells once nano-clays bind with the former through the high non-specific adhesion characteristic of the cancer cells. As the data suggest an important role of nano-clays as an inhibitor of melanoma cell proliferation and survival, these prove to be a natural and effective medicine for the treatment of melanoma. The proven compatibility of nano-clays with the human cells with little side-effects makes them a highly preferred choice for the treatment of melanoma and probably other types of cancers.

## Introduction

Melanoma, although less common but due to being most deadly among skin cancers, accounts for the vast majority of skin cancer death^1^. Recently, an estimate shows that there would be 96,480 new cases of melanoma in the United States and 7230 deaths from the disease^1^. Unlike many forms of cancer, the incidence of melanoma has increased over the last two decades^2^. Current conventional therapies include excisional surgery, immune therapy, and targeted therapy^3,4^. However, there are multiple challenges associated with these treatment options, including tumour size and location, the specificity of medicines, tumour resistance, and adverse side effects. Although there exist specific drugs (such as vemurafenib and dabrafenib) that decrease tumour burden, these are effective only for a limited period^5^. Even as regards combination drug therapy, there are still several critical questions relevant to which combination(s) are most effective. Although, the discovery of processes underlying the traditional immune suppressive features of advanced melanomas has led to essential progress in using immune checkpoint therapies^5^, several immune checkpoint inhibitor drugs, available these days, have got several side effects. The use of natural materials, such as nano-clays, could be one of the alternatives for overcoming several challenges associated with the several available options for the melanoma treatment.

Nano-clays are nano-sized natural materials originating from minerals of the sedimentary rocks. Smectites and palygorskite are two main groups of the nano-clay minerals. Due to isomorphous substitution in their molecular structure, these nano-clays exhibit charge deficiency on their surfaces. This charge deficiency on their surfaces is neutralized by the water molecules and the dissolved cations (Fig. S1). Through considerable research by the authors, we have developed basic characterization and behavior modelling of the charged clay minerals^6–8^. In biomedical field, nano-clays have been successfully used after incorporating these into the cancer medicines including 5-fluorouracil and Trastuzumab^9–15^. Nano-clays can, therefore, be categorized as an alternate medicine for several ailments^16–20^. Clay nanoparticles, being sticky in nature, have been a part of several sustained-release medicines^21,22^; researchers^23^ used anionic clays to be sandwiched with an anti-cancer drug, Methotrexate (MTX). These researchers used the methods based on co-precipitation followed by the hydrothermal action for the preparation of a stable and sustained-release drug-nano clay hybrid. Another group of researchers^24^ bentonite clays could be incorporated in the drugs as delivery vehicles owing to their compatibility and high loading capacity. Resultantly, they prepared a sustained-release delivery system consisting of doxorubicin and nano-clay complex for the treatment of melanoma during chemotherapy. Furthermore, researchers^25^ also studied Montmorillonite clay as drug carrier material in the controlled release of medicine. Some other researchers^26^ also found that nano-clays are quite effective to be used in drug delivery systems and it helps by increasing the efficiency of the drug delivery and hence reducing the toxicity of some of the medicines for the treatment of the thyroid cancer. There are other studies on Halloysite nano-clays that prove the compatibility of these natural materials with the healthy human tissues. Naumenko and Fakhrullin^27^ concluded in their studies that Halloysite nano-clay is biocompatible with the human healthy tissues. Tarasova et al., in their study^28^ also concluded that during their experimentation they found that the pristine Halloysite caused no changes in the morphology of the human cell nuclei. General configuration and the associated physicochemical properties of these nano-clays are provided in the baseline studies^29^ and the supplementary material.

In a previous research^30^, authors showed high affinity of the clay nanoparticles to the other charged particles and surfaces. A combined effect of high affinity of the nano-clays and the high non-specific adhesion property of the cancer cells makes the former highly effective in controlling the tumour metastasis. In that study, we demonstrated that an optimum mix of two charged clay minerals (Na-montmorillonite and palygorskite) could be used for the control of cancer metastasis. Considering these findings^30^, we further hypothesized that nano-clays could also have an inhibitory effect on the cancer cell proliferation and viability. To test this hypothesis, we conducted cell proliferation and cell viability analyses in melanoma cells in vitro and melanoma growth in vivo human melanoma xenograft animal models. Both the in vitro and in vivo studies were conducted at the laboratories of the School of Medicine at the University of Miami, Florida, USA. To substantiate the most probable reasons for the inhibition in the cell proliferation and necrosis, adhesion measurements among healthy and cancerous cells, without and with clay nanoparticles, was also carried out using Atomic Force Microscopy (AFM). Scanning Electron Microscopy (SEM) was also used to visualize the physical interactions of the nano-clays with the cancer cells.

## Materials and methods

### In vitro study

In vitro studies were carried out on human malignant melanoma cells. These cells were exposed to various doses of clay nanoparticle formulations, and their growth and vitality were studied. These doses of nano-clay mix consisting of Na-montmorillonite and Palygorskite were studied and finalized as a proportion of 75:25 in the authors’ previous research^30^.

#### Materials

##### Cell culture

Human malignant melanoma cell line SK-Mel-28 (skin primary melanoma) were obtained from American Type Culture Collection (ATCC, Manassas, VA), while Human Epidermal Melanocytes (HEMs) (adult primary normal cells) were purchased from Cell Applications, Inc. (San Diego). The cells were grown in Dulbecco’s Modified Eagles Medium (DMEM). DMEM was supplemented with 5% Fetal Bovine Serum (FBS) (HyClone Inc.) and antibiotic–antimycotic of 100 μg/ml penicillin, 100 μg/ml streptomycin, and 0.25 μg/ml amphotericin B (Mediatech, Manassas, VA) (DMEM growth media). Cells were maintained in a Thermo culture incubator at 37 °C and 5% CO_2_.

##### Clay nano-particles isolation and purification

Na-montmorillonite (SWy-3) and palygorskite (PFl-l) clay samples were acquired from the Clay Minerals Society (CMS)^29,31^ (Tables S1 to S3). Nano-sized particles of pure clay minerals were obtained by a multi-step procedure involving the deflocculation of the initial dry powdered state and the removal of non-clay minerals and impurities. Most of the steps followed the general guidelines provided in ASTM D7928^32^. First, nanoparticle aggregates in each clay sample were broken down by mixing 5 g of sample with 12.5 ml deflocculant solution (40 g sodium hexametaphosphate in 1000 ml of deionized water) and leaving for 16 h for uniform absorption. Next, the mixture was diluted to 100 ml using deionized water and centrifuged in a standard cup at a speed of 1000 rpm for 30 min. The supernatant was poured into a glass cylinder and further diluted to 1000 ml. The mixture then stood for sedimentation under gravity to remove bigger aggregates and non-clay particles for 12 h. The upper 500 ml portion of the colloid suspension was then extracted.

##### Clay nano-particles size and purity analysis

The clay colloid size distributions were analyzed by the diffraction laser system (DLS). We used a Microtrac S3500 DLS instrument, comprising a Tri-laser system operating at 780 nm wavelength and capable of detecting particle sizes in the range of 0.02–2.8 mm. The run time of each analysis was less than 60 s. The data acquired in the test consisted of particle sizes and their frequency in the suspension. This data was converted by the acquisition software (FLEX) of the instrument to a statistical distribution in the form of the particle size and the corresponding per cent passing. The statistical data is then converted to percentile and similar particle size.

Size distributions were determined both in deionized water and RPMI 1640 medium (Fig. S2). The purpose of doing particle size distribution in deionized water was to determine the maximum possible dispersion of the particles. The resulting finest gradation of each of the clays in deionized water was then compared with the one obtained in the RPMI medium. The purpose was to assess the effect of the presence of salts in RPMI on the flocculation/agglomeration tendency of the clay particles.

After DLS testing, the dry sample of each of the clays was recovered from the colloidal form by evaporating the mixture in an oven at 110 °C for 24 h. The purity of the recovered clay mineral was assessed by X-ray diffraction (XRD) and is shown in Fig. S3.

##### Nano-clays suspension preparations

Based on the previous study by authors^30^, an optimum dose of a combination of nano-clays was carried out (details are provided in the supplementary materials and Figs. S5 and S6). Based on the results of the optimization, stock solutions of SWy-3, PFl-1 and their mix (75:25) was prepared by adding 20 mg of each clay nanoparticles to 10 ml phosphate-buffered saline (PBS) (2 mg/ml in PBS), centrifuged at 25 kpm for 1 h and then filtered through 0.2 μm filter. For in vitro analysis, the stock solution was further diluted in Dulbecco’s modified Eagle’s medium (DMEM) (Lonza, Walkersville, MD) to create 100 μg/ml working solution. For in vivo treatment, the stock solution was diluted in PBS to a concentration of 200 μg/ml.

#### Interaction of nano-clays with human melanoma cells

##### 3-Day dosage response and cell proliferation and viability analyses

For the purpose, 4 × 10^4^ SK-Mel-28 human melanoma cells were plated in each well of a 24-well tissue culture plate (Corning Inc.) and incubated in DMEM growth media overnight at 37 °C and 5% CO_2_. Cells were divided into four groups then treated with control media or treatment media at final concentrations of 2 μg/ml, 5 μg/ml, and 10 μg/ml of respective clay suspensions in DMEM growth media. On day 3, after treatment, cells were washed with PBS, treated with 0.05% trypsin/0.53 mM EDTA solution for 2 min, and detached from the dish. Viable cells and total cells were counted under a phase-contrast microscope with a dye-exclusion hemocytometer technique. Cell viability was calculated by dividing the number of viable cells by the number of total cells (viable cells and non-viable cells). The results were recorded and graphed as a function of treatment concentration versus mean viable cells or cell viability. All treatments were performed in triplicate.

##### 5-Day timing course of cell proliferation and viability assays

For the purpose, 8 × 10^4^ SK-Mel-28 human melanoma cells were plated in each well of a 12-well tissue culture plate. The cells were incubated in DMEM growth media overnight at 37 °C and 5% CO_2_. Cells were divided into four groups then treated with the 10 μg/ml of PFl-1, SWy-3, and mix of PFl-1 and SWy-3 (25:75), respectively, or normal growth media as control. On days 1, 3, and 5 after treatment, melanoma cells were detached and counted using the dye-exclusion hemocytometer technique. The viable cells were counted for cell proliferation, and cell viability was calculated by dividing the number of viable cells by the number of total cells. The results were recorded and graphed as a function of time versus the mean number of viable cells or cell viability. All treatments were performed in triplicate.

#### Interaction of nano-clays with human melanocyte cells

##### 5-Day timing course of cell viability assays

For the purpose, 8 × 10^4^ HEM melanocyte cells were plated in each well of a 12-well tissue culture plate. The cells were incubated in DMEM growth media overnight at 37 °C and 5% CO_2_. Cells were divided into two groups and then treated with the 10 μg/ml of a mix of PFl-1 and SWy-3 (25:75) or normal growth media as control. On day 5 after treatment, the viable cells were counted for the cell proliferation, and cell viability was calculated by dividing the number of viable cells by the number of total cells. Trypan Blue dye was used to differentiate between dead and live cells. The treatments were performed in triplicate.

#### Atomic force microscopy (AFM)

In this study, AFM was used to measure the adhesion among cell–cell and cell-clay configurations. AFM measurements were carried out using an Asylum Research MFP-3D-BIO AFM (Goleta, California, US) mounted on a Nikon A1 confocal microscope at the Miller School of Medicine, University of Miami, Florida. All measurements were carried out at room temperature (25 °C) at a scan velocity of 2 μm/s. The force measurements were carried out using a Veeco MLCT-O10 tipless cantilevers (Camarillo, California, US) with a nominal spring constant of 0.01 N/m. AFM adhesion measurements were conducted using the principle schematically shown in Fig. S4a and S4b and further detailed in the supplementary material. The measurements were performed on four different configurations, i.e., melanocytes (HEM) and melanoma (SK-Mel-28) cells without and with clay nanoparticles.

#### Scanning electron microscopy (SEM)

Cancer cell samples with the nano-clays were imaged in SEM mode in an FEI ESEM-FEG XL-30 at the Miller School of Medicine, University of Miami, Florida. Before SEM imaging, samples were preserved in 2% glutaraldehyde fixative in PBS buffer and stored in the refrigerator for at least 2–3 h. Samples were washed in three changes of PBS buffer for 10 min each and were then post-fixed in 1% osmium tetroxide in PBS buffer for 45 min and then rinsed in three changes of PBS buffer for 10 min each. The cells were then dehydrated in a graded series of ethanol (20, 50, 70, 95, and 100%). After dehydration, samples were dried in three changes of HMDS and left to outgas overnight. Cells on coverslips were then placed on aluminum stubs covered with carbon adhesive tabs. Pelleted cells were placed directly on the carbon adhesive tabs on the stubs.

### In vivo study

#### Animals and facility

Female Nude/Nude mice, 7–8 weeks old, were purchased from Charles River Laboratories (Wilmington, MA). Mice were housed in the Pathogen-free Animal Facility at the University of Miami Miller School of Medicine in RMSB building room #7123 specific for immune-deficient mice or rats, at room temperature (70 ± 1°F) and subjected to a 12 h light/12 h dark cycle. Mice were acclimatized for 1 week before the study; three to four mice were housed in cages to provide adequate space.

Animal protocol (18‐159‐LF) was approved by the Institutional Animal Care and Use Committee (IACUC) of the University of Miami before the experiments. All animal experiments conducted were performed in accordance with the regulations of the American Association for the Accreditation of Laboratory Animal Care (AAALAC). All animal studies were carried out following NIH and Arrive guidelines using University of Miami IACUC-approved protocols.

#### Human melanoma xenograft model

##### Tumour cell injection

Once melanoma cells SK-Mel-28 reached 95–100% confluently, they were washed with PBS, treated with 0.05% trypsin / 0.53 mM EDTA, and detached from the dish. The dye-exclusion hemocytometer technique was used to count viable cells to prepare tumour cell injections. 1 × 10^7^ SK-Mel-28 melanoma cells were resuspended in 200 μl of DMEM for each tumour injection. After anaesthetizing the mice with Isoflurane, melanoma cells were subcutaneously injected into the left and right flank of the nude mice. A total of 50 mice were used for the tumour injections; ten mice were used as normal control without the injection.

##### Nano-clay treatment

Ten days after tumour cell injection, a various number of mice with tumours were chosen for the treatments. Each tumour site was injected with 200 μl of a mix of PFl-1 and SWy-3 (0.2 mg/ml, 25:75) in PBS. Then mice were grouped for the treatment as the following:

- One time on the initial treatment day: 10 tumours/mice
- Weekly for 4 weeks: 10 tumours/mice
- Twice a week for 4 weeks: 10 tumours/mice
- Daily for 3 weeks: 12 tumours/mice
- Tumour control (with tumour injection, without treatment): 12 tumours/mice
- Normal control: no tumour injection and no treatment: 10 mice

##### Tumour tissue collection

At the end of treatment, animals were sacrificed with CO_2_; the tumours were collected immediately by excisions. At the same time, the tumour number was counted; each tumour volume and weight was measured. Tumour weight was recorded in mg using a Scientech 2SA 80 digital scale (Boulder, CO). The length, width, and height of tumours were measured using a Thermo Fisher Scientific digital calliper (Model No. 14-648-17; Tampa, FL). Tumour volume was then calculated as length × width × height.

##### Tissue preparation and histopathology analysis

Excised tumour tissues were fixed in 10% phosphate-buffered formalin, embedded in paraffin, processed to 4 μm thick sections, and stained with Hematoxylin and Eosin (H&E). Tissue slides were then examined under a Zeiss AxioVert 200 M microscopy with a digital imaging system. The morphology of the tissues was evaluated.

The number of cells undergoing mitosis (mitotic figures indicating proliferation) was observed, and ten random fields, at 200 magnification, per group were photographed with AxioCam using the AxioVision 4.6 software (https://www.micro-shop.zeiss.com/en/us/system/software+axiovision-axiovision+program-axiovision+software/10221/). The mean number of dividing cells per field in each group was counted and graphed.

#### Statistical analysis

GraphPad Prism 8 program was used for statistical analysis. A one-way analysis of variance (ANOVA) was used to identify a difference followed by Student *t* tests. P-values less than 0.05 were considered statistically significant.

## Results

### In vitro study

#### Cell proliferation and cell viability in a dosage-dependent manner in 3-day dosage response assay

**Figure 1:**
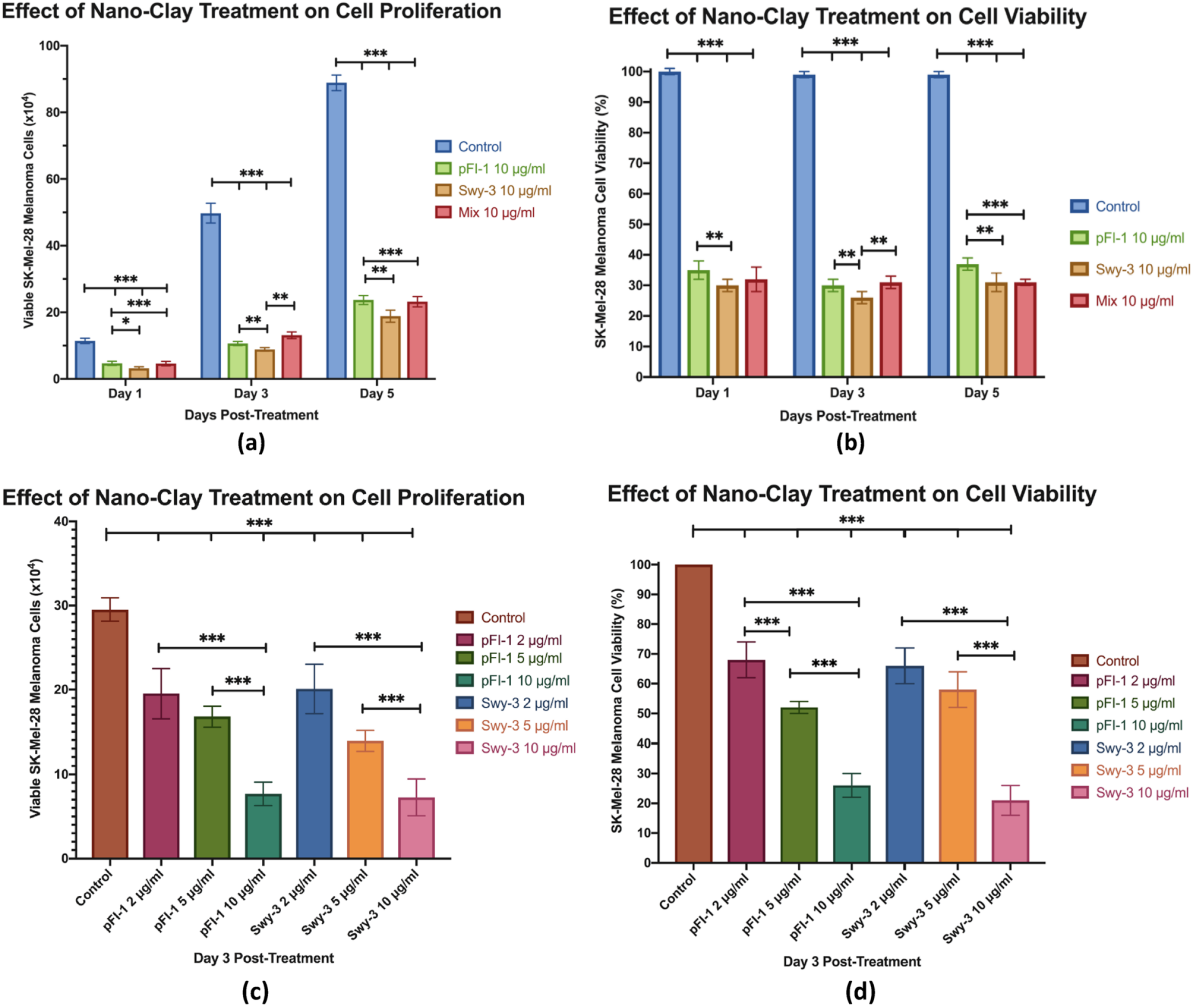
Nano-clay treatments inhibited melanoma cell during a 5-day timing course (**a**) proliferation and (**b**) viability. Treatments results after 3-days treatment, (**c**) reduced melanoma cells, and (**d**) reduced melanoma cell viability in a dosage-dependent manner. SK-Mel-28 melanoma cells were treated with PFl-1, SWy-3 and the mix (25:75) at concentrations of 0 (control), 2, 5 or 10 μg/ml for 5 days. The numbers of viable cells were counted at days 1, 3, and 5 post-treatment, and viable cell numbers for the treatments were presented as columns. Standard deviation is represented in the figure by a bar attached to each column. Significant differences at p < 0.05, p < 0.01 and p < 0.001 are denoted by *, ** and ***, respectively.

Three days after treatment, the control group melanoma cells exhibited the greatest number of cells and cell viability (100%). In contrast, the viable cells and cell viability in all treatment groups were significantly decreased (Table S4, Fig. 1a,b). Furthermore, the viable melanoma cell numbers were significantly reduced in a dosage-dependent manner when the concentrations of the treatment of both PFl-1 and SWy-3 increased from 2 to 5 μg/ml, and 10 μg/ml (Table S4, Fig. 1). Similarly, the viability of melanoma cells decreased markedly in a dosage-dependent manner as the treatment concentrations of both PFl-1 and SWy-3 increased (Table S4, Fig. 1b).

The viability of melanoma cells treated with PFl-1 at concentrations of 2 μg/ml, 5 μg/ml, and 10 μg/ml were 68%, 52%, and 26%, respectively (Table S4, Fig. 1b). The decrease was significant compared to the control (all p < 0.0001). Furthermore, cells treated with the 10 μg/ml concentration of PFl-1 resulted in the greatest decrease in viable cell count and cell viability (26%) amongst all PFl-1 treatment groups (Table S4, Fig. 1a,b) (p < 0.0001).

The viabilities of cells treated with SWy-3 at concentrations of 2 μg/ml, 5 μg/ml, and 10 μg/ml were 66%, 58%, and 21%, respectively (Table S4, Fig. 1b). The decrease was significant as compared to the control (all p < 0.0001). Furthermore, cells treated with 10 μg/ml of SWy-3 resulted in the greatest decrease in mean viable cells and viability (21%) amongst all SWy-3 treatment groups (Table S4, Fig. 1a,b) (p < 0.0001).

#### Melanoma cell proliferation and viability in a 5-day timing course analysis

The results of a 3-day dosage response assay led us to further study cell proliferation effects in a 5-day timing course with both nano-clay treatments at a concentration of 10 μg/ml. The 10 μg/ml concentration was chosen as it inhibited cell proliferation and viability the most.

There were significant decreases in mean viable cell counts and viabilities at all three timing points when comparing the 10 μg/ml concentration of PFl-1 treatment to the Control (Table S5, Fig. 1c,d).The viabilities of melanoma cells were 35%, 30% and 37% at days 1, 3 and 5, respectively, post-treatment (p < 0.0001). Furthermore, there was a significant decrease in mean viable cell counts and viabilities at all three timing points when comparing the 10 μg/ml concentration of SWy-3 treatment to the control. The viabilities of melanoma cells were 30%, 26% and 31% at days 1, 3 and 5, respectively, post-treatment (Table S5, Fig. 1c,d) (p < 0.0001). Lastly, there was a significant decrease in mean viable cell counts and viabilities at all three timing points when comparing the 10 μg/ml concentration of mix of PFl-1 and SWy-3 (25:75) treatment to the control. The viabilities of melanoma cells were 32%, 31% and 31% at days 1, 3 and 5, respectively, post-treatment (Table S5, Fig. 1c,d) (p < 0.0001).

#### Melanocytes cell viability in a 5-day timing course analysis

**Figure 2:**
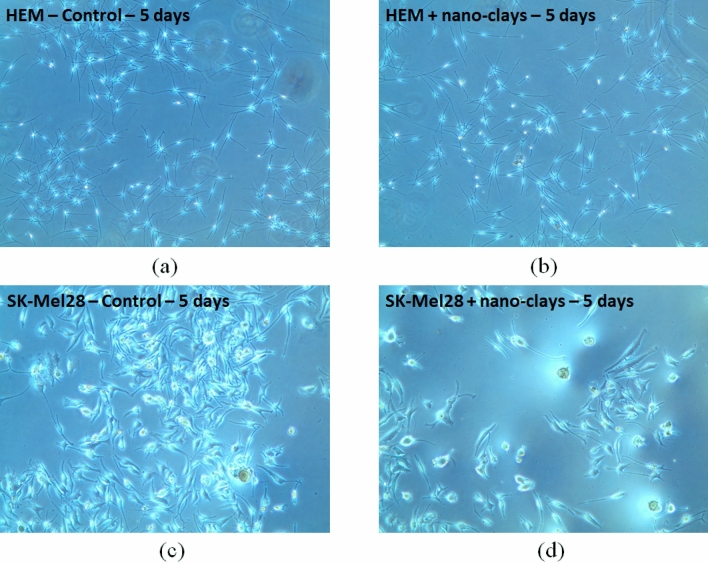
Viability of melanoma and melanocytes cells using nano-clays (**a**) and (**b**) respectively control and 5-days after nano-clay treatment of melanocyte (HEM) cells, (**c**) and (**d**) respectively control and 5-days after nano-clay treatment of melanoma cells (SK-Mel-28). The cells were treated with PFl-1 and SWy-3 mix (25:75) at concentrations of 0 (control) and 10 μg/ml for 5 days. The numbers of viable cells were counted at day 5 post-treatment.

Essentially, all of the Human Epidermal Melanocyte (HEM) appeared viable even after 5 days in culture with the clay particles (Fig. 2). There might be a couple of dead cells per slide (Fig. 2b), but almost all of the cells look really healthy. By contrast, many SK Mel-28 cells were rounded up and dying after days in clay (Fig. 2d).

### In vivo tumour xenograft study

#### Effects on tumour volume

**Figure 3:**
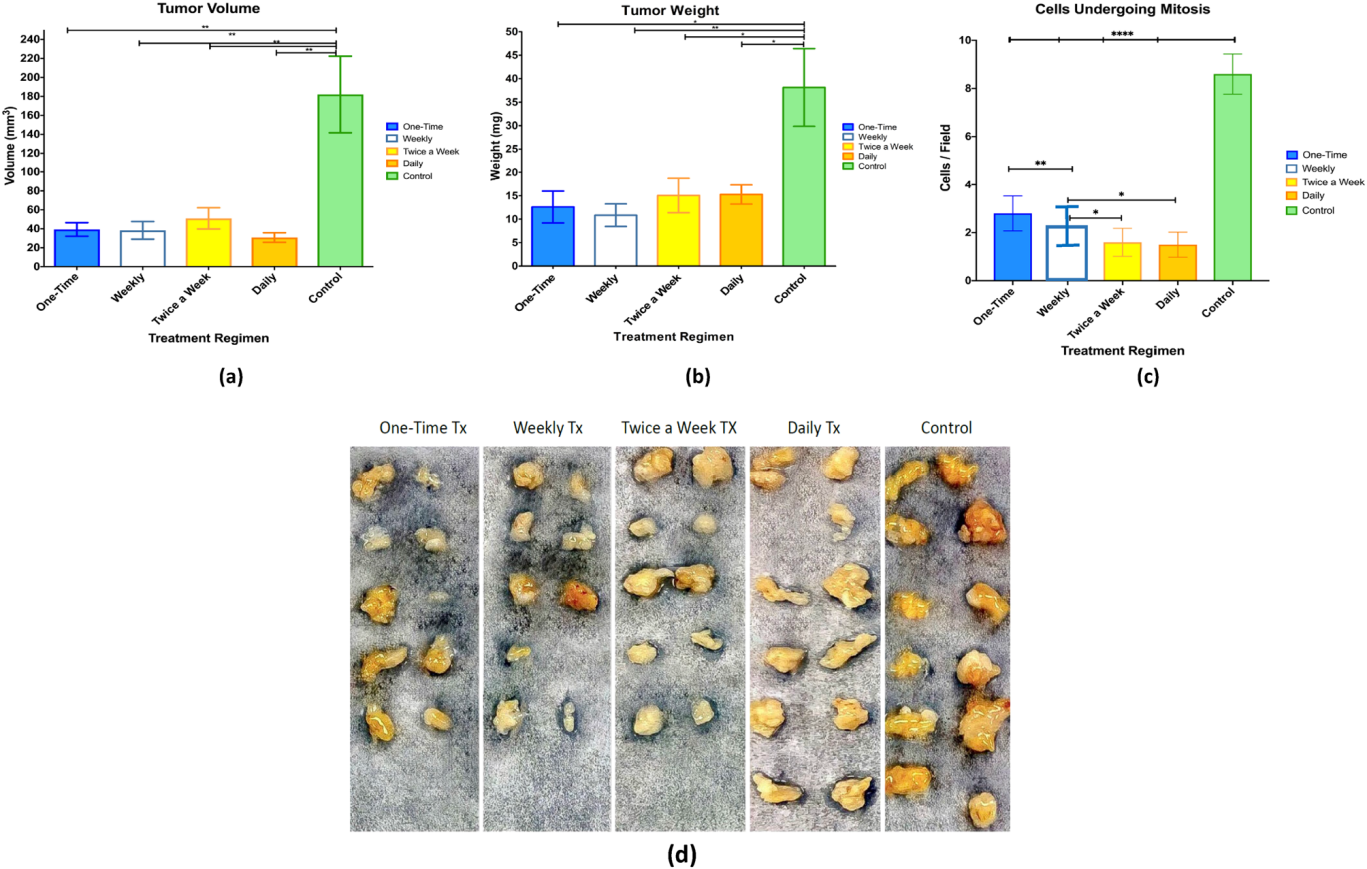
Nano-clay treatment regimens. (**a**) Reduced tumour volume, (**b**) reduced tumour weight, and (**c**) reduced number of cells undergoing mitosis. The standard error is represented in the figure by a bar attached to each column. Significant differences at p < 0.05 and < 0.01 are denoted by * and **, respectively. (**d**) Tumours were photographed after tumours were excised and presented as treatment groups.

The tumour volumes in all treatment groups were significantly decreased compared with the untreated control tumour group (Table S6, Fig. 3a,d). The mean tumour volume for the groups treated one time on initial treatment day, weekly for 4 weeks, twice a week for 4 weeks, and daily for 3 weeks were 39.31 mm^3^, 38.36 mm^3^, 50.99 mm^3^, and 30.79 mm^3^, respectively. The decrease was significant as compared to the untreated control tumours, which exhibited the highest mean volume of 182.02 mm^3^ (all p < 0.01). Furthermore, the tumours treated daily resulted in the maximum decrease in size (Table S6, Fig. 3a). No significant differences were measured among treatment groups.

#### Effects on tumour weight

The tumour weights in all treatment groups were significantly decreased compared to untreated controls (Table S6, Fig. 3b). The mean tumour weights for tumours treated one time on initial treatment day, weekly for 4 weeks, twice a week for 4 weeks, daily for 3 weeks and untreated controls were 12.60 mg, 10.86 mg, 15.04 mg, 15.27 mg, and 38.14 mg, respectively (Table S6, Fig. 3b). The tumours with daily, twice a week, and one-time treatment regimens demonstrated a significant decrease compared to the untreated controls (all p < 0.05). In contrast, tumours treated weekly resulted in the maximum reduction in weight compared to the control (p < 0.01) (Table S6, Fig. 3b). There were no significant differences among treatment groups.

#### Effects on tumour cell mitotic figures

Mitotic figures present malignant tumour cells of dividing. Untreated tumours exhibited the highest number of cells undergoing mitosis; the mitotic counts in all treatment groups were significantly decreased, while tumours treated weekly showed no mitotic figures (Table S7, Fig. 3c,d). The mean mitotic counts per high power field (200 magnification) for tumours untreated controls, treated one time, weekly, twice a week, and daily were 8.60, 2.80, 2.00, 1.60, and 1.50, respectively (all p < 0.0001) (Table S7, Fig. 3c). The difference among treatment groups existed between weekly and one-time treatment (p < 0.01), weekly and twice a week treatment (p < 0.05), and weekly vs daily treatment (p < 0.05).

#### Tumour necrosis and inflammatory cell infiltration

**Figure 4:**
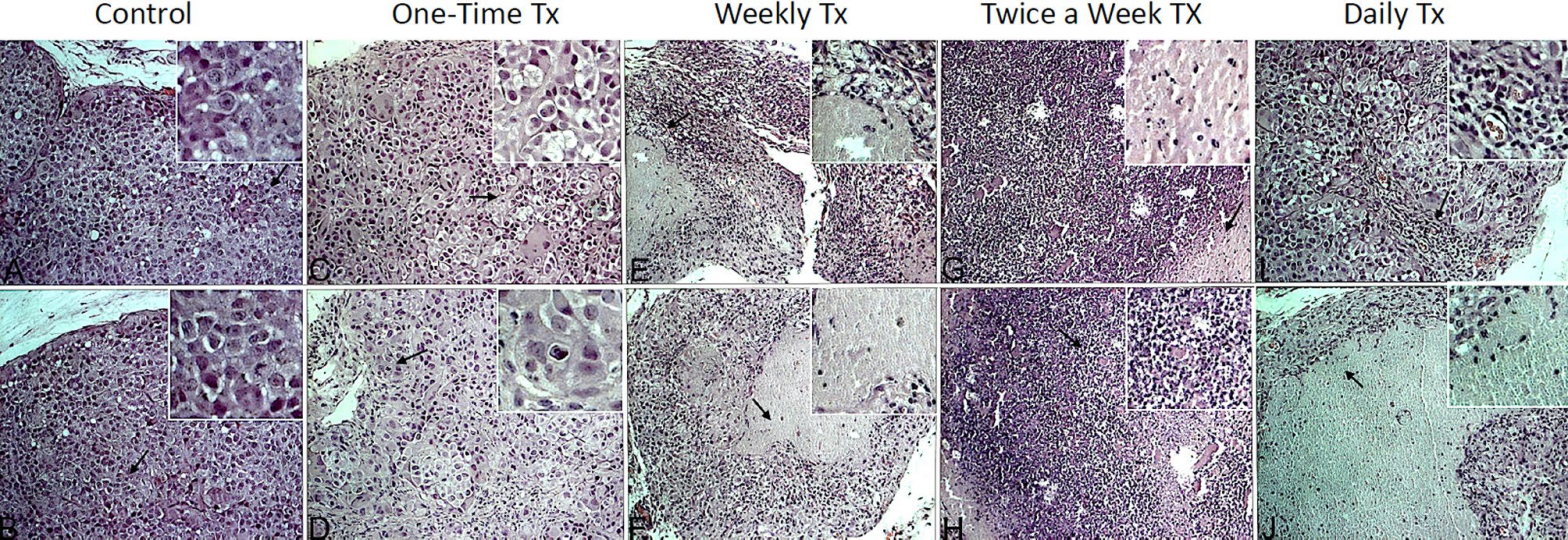
Melanoma tissue histology images of H&E staining. Some representative tumour images were photographed. Each insertion of magnified areas in the top right corner, highlighting some important figures. Arrows point to the areas of insertion. (**A**,**B**) Untreated (control) tumours demonstrated multiple cells undergoing mitosis, with no inflammation or necrosis. The magnified insertion of (**A**) shows cells with condensed chromatin, most likely in the prophase of mitosis. The magnified portion of (**B**) shows multiple cells with condensed chromatin, and two cells recently underwent mitosis. (**C**,**D**) Tumours treated one time during the initial treatment demonstrated cells undergoing mitosis, with some inflammatory cell infiltration. The magnified insertions of both (**C**,**D**) show cells with condensed chromatin. (**E**,**F**) Tumours treated weekly for 4 weeks demonstrated very few cells undergoing mitosis. The magnified insertion portion of (**E**) shows white cell infiltration around necrotic tissue, while the magnified insertion of (**F**) highlights necrotic tissue. (**G**,**H**) Tumours treated twice a week for 4 weeks showed significant white cell infiltration and some necrosis. In the magnified portion of (**G**), some necrotic tissue with cellular infiltrate is present, while the magnified portion of (**H**) shows cellular infiltration. (**I**,**J**) Tumours treated daily showed significant necrosis and white cell infiltration. The infiltration around blood vessels can be seen in the magnified portion of (**I**), while the infiltration in necrotic tissue is highlighted in (**J)**.

As shown in Fig. 4, the histopathological analysis demonstrates a large amount of tumour cell death in nano-clay treated tumours. There were marked tumour tissue necrosis in tumours treated weekly (Fig. 4E,F), twice a week (Fig. 4G,H), daily (Fig. 4I,J), and even one time (Fig. 4C,D) compared with untreated controls (Fig. 4A,B). Tumours treated weekly were significantly smaller and accompanied by a large area of tissue necrosis that was surrounded by inflammatory cell infiltration (Fig. 4E,F). Tumours treated daily exhibited the highest areas of necrosis. Untreated tumours differ significantly from all treatment groups as there was no necrosis and with minimal cell infiltration.

## Discussion

The results obtained from these studies indicate that nano-clays inhibit melanoma cell growth via inhibiting cell proliferation and cell survival. Similarly, the viability of melanoma cells markedly reduced in a dosage-dependent way for both types of nano-clays. Histological analysis of multiple fields of tumours in each group showed that all treatments significantly inhibited tumour cell mitosis. Furthermore, histological examination revealed marked necrotic tissue in tumours with nano-clay treatment while there was no tumour necrosis in untreated tumours. These in vivo findings are consistent with the in vitro data that clay nanoparticles reduced melanoma cell growth probably by inhibiting cell proliferation and cell survival or inducing cell death. The possible mechanisms by which clay nanoparticles are causing inhibition of the cancer cell mitosis have been discussed in the light of the interactions of these nano-clays with the tumour microenvironment.

### Nano-clays’ interactions with the tumour microenvironment

Based on the evidence and analysis of the current study and authors’ experience with the nano-clays from their earlier studies, the nature of the probable interactions of the nano-clays with cancer cells could be essentially divided into two parts; physical and biophysical. Primarily, charged nano-clays surfaces interact with cancer cells using the high non-specific adhesion characteristic of the later, leading to a complete binding, enveloping, and bridging of the cancer cells. In addition to the physical interaction of the cancer cells and nano-clays, there are certain biophysical changes in the cancer cell surfaces leading to the inhibition of cell proliferation and necrosis.

#### Physical: adhesion of nano-clays through non-specific adhesion of cancer cells

**Figure 5:**
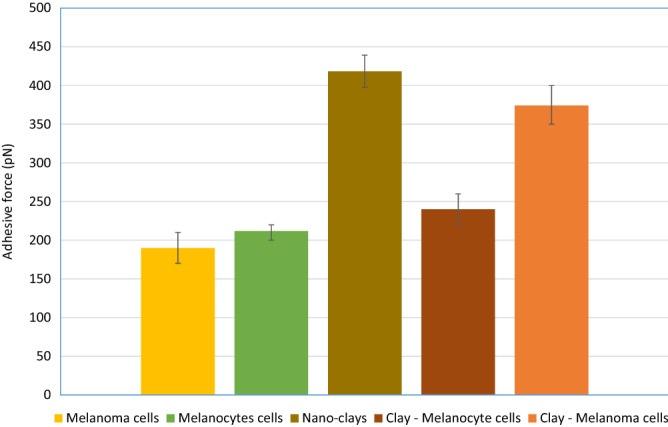
Summary of adhesion force measurements among melanoma and melanocyte cells using AFM, before and after treatment with clay nanoparticles. Error bars represent the variations in adhesive force in three trials.

**Figure 6:**
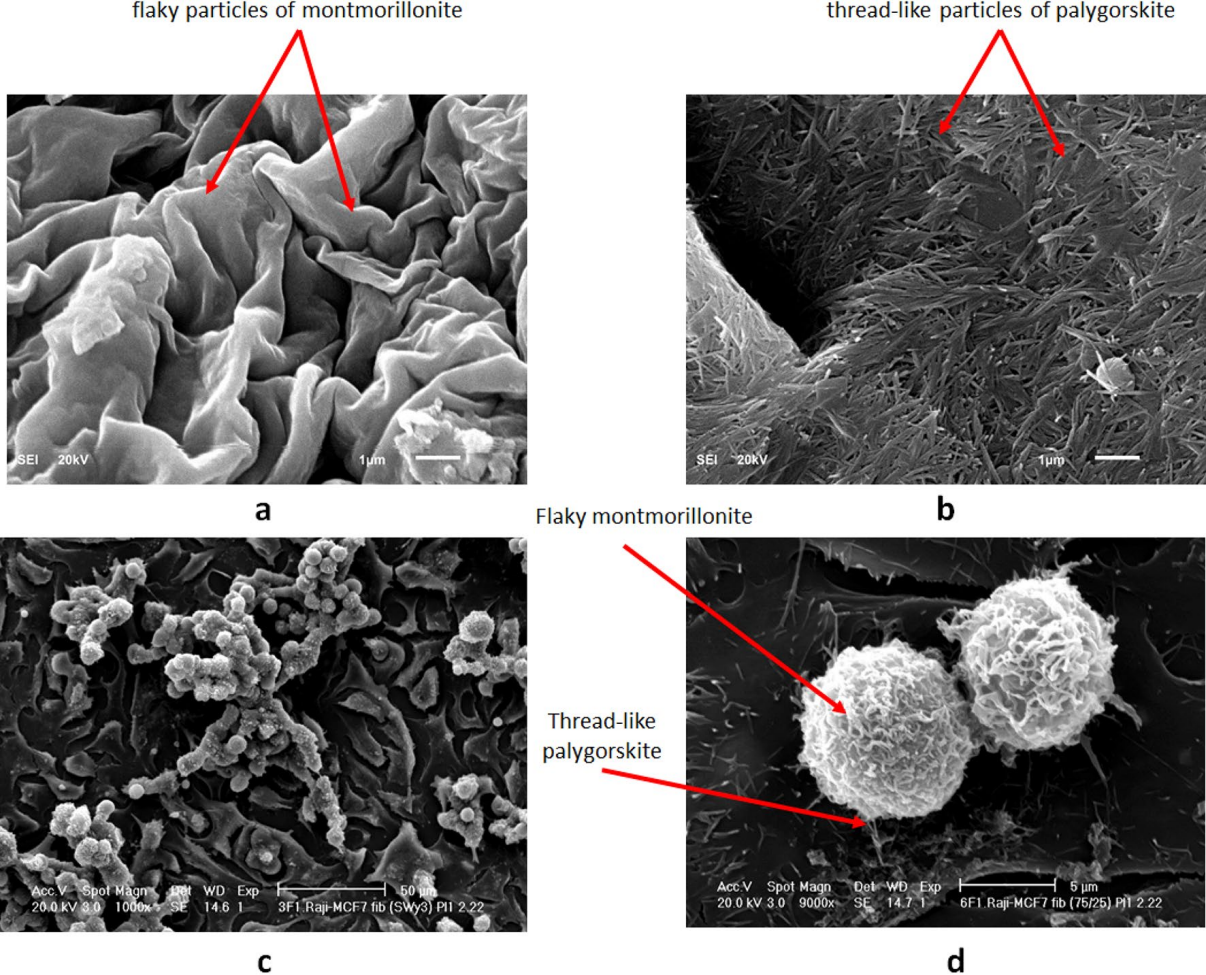
SEMs of the nano-clays and cancer cells interaction. (**a**) Na-montmorillonite flaky particles. (**b**) Thread-like Palygorskite particles. (**c**) Na-montmorillonite/Palygorskite particles enveloping/binding the multiple cancer cells. (**d**) Close-up view of couple of cancer cells enveloped by the nano-clays.

Authors have demonstrated the role of nano-clays in promoting adhesion among the cancer cells and their microenvironment and hence controlling metastasis^30^. In the current study, the adhesion measurements among cancer/healthy cells and nano-clays using AFM revealed a marked increase in adhesion (~ 100%) when nano-clays were added to the melanoma cells. In contrast, no significant increase in adhesion was observed in the case of healthy melanocyte cells (Fig. 5). Many previous studies including^33^ have concluded that healthy human cells bear higher specific adhesion mediated primarily by cadherins and integrins. But when these cells become cancerous, they lose specific adhesion while gain higher non-specific adhesion. This non-specific adhesion in cancer cells originates from the two sources i.e., van der Waals and electrostatic forces and the negative charges on their surface as compared to normal cells. Among these non-specific adhesion forces, van der Waals are the most contributing, while electrostatic ones are least contributing and may be affected by the presence of the salts in the medium^34^. Several other similar studies^35–38^ have also indicated that due to the secretion of lactate ions and sialic acid, the positive ions from the cell surface move to the intracellular space, resulting in the negative charges on the cell surface. Cancer cells, due to these specific characteristics, become highly suitable candidates to be bonded by the clay nanoparticles. SEMs of the cancer cells interactions with clay nanoparticles in Fig. 6 reveal a complete enveloping of the cancer cells with the nano-clays and it is also a testimony of the high binding attractions among each other. On the other hand, as evidenced in Fig. 5, healthy cells, due to the absence of the non-specific attractions, do not promote such adhesions with the nano-clays. So, as cancer cells bear high negative charges, loss of specific adhesion, and gain of non-specific adhesion, melanoma cells facilitate adhesion with the charged nano-clays. In contrast, the absence of any such free adhesion force does not promote any such adherence in case of healthy cells. This also supports the conclusion that other processes leading to the inhibition of cell proliferation and necrosis would also be absent in case of healthy cells, and nano-clays do target cancer cells only. This fact is further explained in the light of the non-toxicity of the clays to healthy human cells in the section on the compatibility of the nano-clays with the healthy human cells.

**Figure 7:**
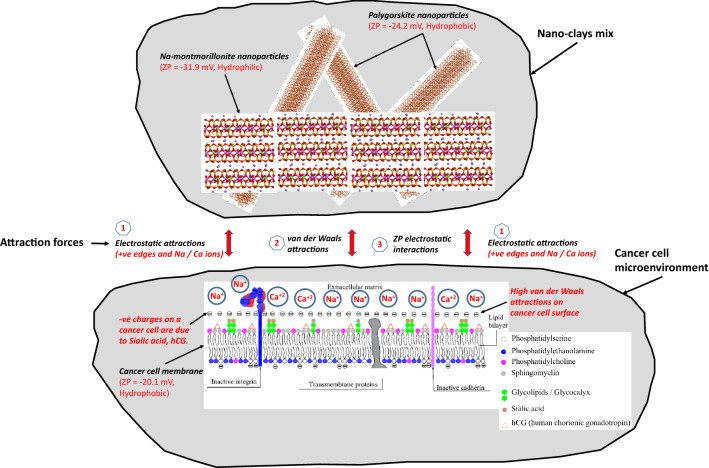
Three possible mechanisms of interactions of montmorillonite-palygorskite mixed clays with the cancer cells: (1) electrostatic attraction among positively charged nanoparticle edges and Na/Ca/Mg ions with negatively charged protein surfaces, (2) van der Waals attractions, (3) ZP electrostatic interactions.

Based on the possible physical interactions, a schematic interaction model of nano-clays and the cancer cells have been prepared and shown in Fig. 7. Authors concluded in their previous study that adhesion processes are facilitated by the van der Waals, electrostatic, and zeta potential (ZP) attractions^30^. Zeta potential is the charge on a particle at its shear plane along the diffuse double layer when present in suspension. It helps in understanding and predicting the interactions between particles during suspension such as dispersion, agglomeration, etc. Generally, if ZP of a material is greater than 30 mV (either positive or negative), it designates its tendency to disperse. On the other hand, a ZP value of lesser than five mV indicates the tendency of material to aggregate in suspension. In that study, the clay nanoparticles demonstrated a high dispersion tendency due to their higher ZP (− 24 to − 32 mV) values. This consequently led to the availability of higher surface areas and hence increasing the interaction probability with the tumour cells. Although considering their ZP values, Na-montmorillonite nanoparticles are categorized as hydrophilic in nature, they generate a secondary adhesion with the hydrophobic cell surfaces in the salt rich environment^39^. According to the baseline studies on these nano-clays (Table S3), they also demonstrate higher dispersion tendencies owing to both the hydrophilic nature and relatively higher acid–base (AB) repulsion forces. This dispersion tendency leads to generation of higher surface area and hence in the increased attractive interactions. Palygorskite due to their higher surface area of 136 m^2^/g, results in greater attractions due to the van der Waal attractions and the electrostatic forces. On the other hand, Na-montmorillonite has a relatively lesser surface area (32 m^2^/g), but they also get attracted to the negative cell surfaces through their positively charged edges. The optimized proportion of Na-montmorillonite and palygorskite (75/25) used in this study primarily owes to the maximum adhesion caused by this proportion (Fig. S6) as compared to the other proportions. Moreover, due to almost same surface area of each of the component at this proportion (75/25) results in the maximization of the contribution of the each type of nano-clay. At this proportion, Na-montmorillonite is providing maximum coating, while palygorskite is resulting in maximum bridging action.

In addition to the above-explained interaction mechanisms, attractions also exist among the dispersed sodium/calcium cations from the clay dispersed as colloids in PBS medium, and the negatively charged cell surfaces. This mechanism results in the reduction of the potential repulsion among the negatively-charged clays and the cancer cells. Reduction in repulsion brings them closer and thus promotes the bonding of the clay particles and the cancer cells.

#### Biophysical: changes in membrane potential of the cancer cells

As established in the above-narrated adhesion mechanism promoted by the multiple forces of attraction (Fig. 7), cancer cells have been completely covered and enveloped by the nano-clays particles (Fig. 6). Now, due to the charged nature and the specific configuration of the nano-clay particles in the post-binding state, certain changes in the membrane potential and other associated characteristics of the cancer cells are anticipated.

Normal cells pass through several transformations in physical and chemical structure that lead these cells to become carcinogenic. Two such typical electrical features of cancer cells are a regularly maintained low membrane potential and the high concentration of the intracellular concentration of sodium^40–42^. Healthy human cells are characterized by high concentration of potassium and a low concentration of sodium, but once these cells become cancerous, sodium and water move inside the cells and potassium, magnesium, calcium and zinc move out of the cell^42,43^. All this phenomena results in lowering the membrane potential of the cancerous cells to about − 15 mV as compared to − 60 to − 100 mV of their healthy counterparts^35^. Now, since the membrane potential of a healthy cell facilitates several processes including the cell membrane transport mechanisms and DNA activity, synthesis of protein, and aerobic energy production, the cancerous cells will suffer lack of the normal metabolic activity due to the electronic conductivity function. Furthermore, the reduction of the potential of the cancer cell membranes cause changes in its permeability and thus hindering the flow of oxygen and nutrients into the cell^33^. Thus all these phenomena consisting of anaerobic metabolism due to lack of oxygen, excessive sodium concentrations inside the cell, lower transmembrane potential, and pH alterations result in the creation of the intracellular conditions leading to the formation of cancerous cells.

Based on all the above, a key consideration in cancer treatment could be to restore the healthy membrane potential in the cancer cells^44,45^. In our case, inhibition of cancer cell proliferation and necrosis could be closely attributed to the restoration of the membrane potential of the cancer cells. The change in the membrane potential could be linked with the reversal of negative charges due to the several attraction forces including the supply of the cations from the clay particles, (Na^+^ and Ca^2+^ in Fig. 7). This also results in the redistribution of the salts present on the cancer cells after the adherence to the nano-clays and hence the restoration of the salt balance of the healthy cells.

### Nano-clays compatibility with human cells

In addition to the confirmation of nano-clays being nontoxic to the human healthy cells (melanocytes) during the current study, nano-clays have also been used successfully as an essential part of several medicines as drug carriers. Moreover, as these nano-clays are also a central part of many slow-release medicines, they have been tested as part of the drug approval experimentation and testing^46^, and concluding no cytotoxicity to human healthy cells. In another study, Kaolinite clay mineral was shown to have high biocompatibility and very low cytotoxicity when used as a drug carrier^9^. In-vitro studies on poly (d,l-lactide-co-glycolide)/montmorillonite nanoparticles complex showed negligible cytotoxicity^12^. In another in-vitro study^11^, palygorskite-polyethyleneimine-fluorescein isothiocyanate nanocomposites demonstrated almost nil cytotoxicity^11^. Considering a safe use of the nano-clays in cancer and other drugs as carriers^23–26^, their proven safety since a prolonged use in many cosmetics and skin applications, nano-clays can be categorized as safe for the healthy human cells. There are other studies on Halloysite nano-clays that prove the compatibility of these natural nano-clays with the healthy human tissues^27,28^. So, we recommend that nano-clays may be used as 'clay-alone' medicine without any undesirable effect on the healthy cells. Moreover, although nano-clays have been classed as non-biodegradable in nature, a comprehensive study on the use of similar inorganic nanoparticles in the body^47^ have proven these nano-clays to be ‘human body clearable inorganic agents’.

## Conclusions

The results of in vitro and in vivo studies have proven that nano-clays could be used as natural and effective medicine for the treatment of melanoma. The nano-clays have been found to interacting with the cancer cells due to the high non-specific adhesion of the later. The physical process of complete binding and enveloping of the cancer cells with the nano-clays through the non-specific adhesion of the former leads to the change in the membrane potential of the cancer cells, which in turn leads to the inhibition of the cell proliferation and necrosis. The change in the membrane potential could be linked with the reversal of negative charges due to the several attraction forces including the supply of the cations from the clay particles. This also results in the redistribution of the salts present on the cancer cells after the adherence to the nano-clays and hence the restoration of the salt balance of the healthy cells.

The biocompatibility of nano-clays with the human cells having little side-effects make them a highly preferred choice for the treatment of melanoma and other types of cancers. Since the interaction of nano-clays with the cancer cells takes place through their non-specific adhesion, no such interaction is obvious due to the absence of the non-specific adhesion in healthy cells.

These encouraging results support the commencement of further pre-clinical or clinical studies to investigate the practical applicability of the approach. Based on the conclusions of the current study, further pre-clinical/clinical research could be planned using 75/25 proportion of Na-montmorillonite and palygorskite.

Based on the findings of the study, other natural or manufactured nanoparticles having similar characteristics as of nano-clays may also be studied for the potential treatment of various types of cancer.

## Supplementary Information

Supplementary Information.
