# Supplementary material for: Melanoma treatment via non-specific adhesion of cancer cells using charged nano-clays in pre-clinical studies
Source: Sci Rep. 2021 Feb 2;11:2737. doi: 10.1038/s41598-021-82441-8 (PMC7854583; doi:10.1038/s41598-021-82441-8)
Supplement: Supplementary file 1 — Supplementary Information. [file 41598_2021_82441_MOESM1_ESM.doc]

**Melanoma treatment via non-specific adhesion of cancer cells using charged nano-clays in pre-clinical studies**

Sahel N. Abduljauwad Ph.D^1^, Habib-ur-Rehman Ahmed Ph.D^1^* and Vincent T. Moy^2^

^1^Civil & Environmental Engineering Department, King Fahd University of Petroleum & Minerals (KFUPM), Dhahran, Saudi Arabia

^2^Department of Physiology & Biophysics, University of Miami, Florida, USA

*Correspondence: habibrehman@kfupm.edu.sa

**Supplementary Material**

**NANO-CLAYS [31]**

Each of the electrically active clay minerals has its distinct morphology, characteristics, and interaction behavior. Na-montmorillonite is a layered phyllosilicate clay smectite. In the colloid form, the space between adjacent layers can contain free sodium, calcium, or magnesium cations that can become electrostatically attracted to external negatively charged surfaces [30]. Owing to the extent of the isomorphous substitution in the clay layers, Na-montmorillonite has net negative charges on its flake-like interlayer surfaces. In its dry powdered state, Na-montmorillonite exists as equidimensional flakes/sheets with dimensions of approximately 0.5 x 0.5 x 0.001 microns (**Fig. S1**). The negative charges on their interlayer surfaces are balanced by the cations. In colloidal form, these cations dissociate from these clay particle surfaces and interact with the other negatively charged surfaces. The flaky particles of Na-montmorillonite exist mostly as equidimensional flakes. These particles also have positively charged edges due to the presence of the broken bonds at their ends.

The individual clay nanoparticles join at the edges and ends to form several times bigger particles and then adhere/coat, and bridge the non-clay mineral particles present in soils to impart adhesion. Similarly, the charged structure and large surface area of clay nanoparticles give them an affinity for charged entities, as found on bacterial surfaces and bacterial toxins. Their biomedical properties of interest include high absorption, ability to engulf microbes, and low toxicity.

As a contrast to smectites, palygorskite has a lathe or thread-like structure with alternating regions of negative and positive charges that allow attraction to external surfaces of either charge. Palygorskite has a net interlayer charge in addition to the positively and negatively charged on alternate edges along the entire outer surface of the thread-like particles (**Fig. S1**). Morphologically, the palygorskite particles are tubular. They also differ from the other layered silicates as they lack continuous octahedral sheets. Due to exposure of oxygen and hydrogen on the alternate edges, alternate negative and positive charges prevail along the outer surface of the tubular structure. This feature is unique to palygorskite and makes it versatile in being attracted to either positively or negatively charged surfaces. Due to the discontinuous octahedral sheets in palygorskite, some octahedral magnesium ions are exposed at the edges and hold bound water molecules (OH_2_). These magnesium ions are also available for electrostatically binding to negatively charged surfaces when the clay is present in the colloid form. In addition to the bound water, variable amounts of zeolitic (i.e., free) water (H_2_O) are contained in the rectangular channels.

**ATOMIC FORCE MICROSCOPY (AFM) MEASUREMENTS**

AFM measurements were carried out using an Asylum Research MFP-3D-BIO AFM (Goleta, California, US) mounted on a Nikon A1 confocal microscope at the Miller School of Medicine, University of Miami, Florida. All measurements were carried out at room temperature (25ºC) at a scan velocity of 2 μm/sec. The force measurements were carried out using a Veeco MLCT-O10 tipless cantilevers (Camarillo, California, US) with a nominal spring constant of 0.01 N/m. AFM adhesion measurements were conducted using the principle schematically shown in **Fig. S4a and S4b**. The measurements were performed on 4 different configurations.

***Melanocytes and Melanoma cells with nano-clays***

Cantilevers were initially salinized with 3-aminopropyltriethoxysilane. After incubation of the cantilevers with 0.1% glutaraldehyde for 30 min, melanocytes (2.5 μg/ml) were coupled to the cantilever through the glutaraldehyde linker. Incubation for 1 h with 1% bovine serum albumin (BSA) was used to block the bare surfaces of the cantilever.

Melanocyte cells were plated on 35 mm Petri dishes (Falcon 351008) that were coated overnight with 0.1 mg/ml poly-L-lysine (Sigma) and returned to the incubator for 30 min to allow the cells to get attached to the surface of the dish. Afterwards, the cells were exposed to clay suspension at the concentration of 0.2 mg/ml. After 30 min, the clay medium was replaced with fresh cell culture medium. With the aid of the light microscope, the end of a fibronectin-functionalized cantilever was positioned on the center of a melanocyte cell. Adhesion measurements were acquired by lowering the cantilever onto the melanocyte cell and withdrawing the cantilever until it detaches from the cell. Measurements were averaged for three trials; each trial in turn consisted of 15 measurements on 15 cells.

***Melanocytes and Melanoma cells without nano-clays***

For cell-cell measurements, the cantilevers were functionalized with concanavalin A (ConA). For the purpose, the cantilevers were soaked briefly in 0.1 M NaHCO3 (pH 9.0) to ionize the surface of the cantilevers, removed from the NaHCO3 solution, air-dried, and immersed in 100 μl of ConA (0.1 mg/ml in PBS buffer) overnight at 4°C.ConA–functionalized tipless AFM cantilevers were then further functionalized with the cells using a glutaraldehyde linkage.

The measurements were made between melanoma cell attached to the end of the AFM cantilever and a melanoma monolayer plated on a tissue culture dish, without and with the clay suspension in RPMI medium at a concentration of 0.2 mg/ml. The measurements were repeated in the absence and in the presence of the charged clays.

***Statistical analysis***

GraphPad Prism 8 program was used for statistical analysis. A one-way analysis of variance (ANOVA) was used to identify a difference followed by Student T-tests. P-values of less than 0.05 were obtained in the analysis and considered statistically significant.

**NANO-CLAYS MIX OPTIMIZATION**

Nano-clays mix was optimized using the results of the particle size analysis, SEMs, and the maximum adhesive force in AFM.

Despite relatively bigger particle sizes of the individual clays, mixtures of Na-montmorillonite and palygorskite have been observed to have much finer gradations (**Fig. S2**). The mixtures have finer gradations even in RPMI medium that would have resulted, otherwise, in bigger flocculated particles. Na-montmorillonite and palygorskite mixtures with 25/75, 50/50, and 75/25 proportions, prepared in RPMI medium, result in mean size of 25, 34, and 49 nm respectively. It could further be observed from **Fig. S2,** that higher the percentage of palygorskite in the mixture, finer is the resulting gradation. SEMs of palygorskite and Na-montmorillonite interacting with cell-cell-FN (**Fig. S5**) also support the fact that there is a predominance of palygorskite thread-like particles in 50/50 and 25/75 Na-montmorillonite/palygorskite mixtures treated cells-FN (**Fig. S5a and S5b**), and all the Na-montmorillonite particles seem to be completely covered by the several layers of palygorskite particles. On the other hand, the resulting particles in the 75/25 proportioned mixture seem to have equal representation of both the clays (**Fig. S5c**).

Adhesion measurements of the mixtures of Na-montmorillonite and palygorskite in proportions of 25/75 and 50/50 reveal slight increase in adhesion (5 and 20% respectively) among Raji cells and FN configuration, while proportion of 75/25 has resulted in an increase in adhesion by 100% (**Fig. S6**). Use of Na-montmorillonite alone without palygorskite on cell-cell-ECM configuration, however, resulted in an increase in adhesion by about 70%. So, maximum increase in adhesion in a Raji cells and FN environment is facilitated by Na-montmorillonite and palygorskite in a proportion of 75/25.

**Table S1: Summary of chemical and physical characterizations of nano-clay samples [32**]

| **Sample Designation** | | **Clay mineral** | **Other minerals** | | | **Surface Area N_2_ (m^2^/g)** | **CEC (meq/100 g)** | **Exchangeable cations** | | **Octahedral charge** | **Tetrahedral charge** | **Interlayer charge** |
| --- | --- | --- | --- | --- | --- | --- | --- | --- | --- | --- | --- | --- |
| **SWy-3** | **Na-montmorillonite** | | | **5% silica** | **31.82** | | **76.4** | | **Na, Ca** | **-0.53** | **-0.02** | **-0.55** |
| **PFl-1** | **Palygorskite** | | | **5% silica** | **136.15** | | **19.5** | | **Mg** | **-1.87** | **-0.22** | **-2.09** |

**Table S2: Summary of the chemical composition of the clay samples [30**]

| **Sample Designation** | | **Major clay mineral** | **Source** | **Chemical formula** |
| --- | --- | --- | --- | --- |
| **SWy-3** | **Na-montmorillonite** | | **Crook County, WY, USA** | **(Na,Ca)_0.33_(Al,Mg)_2_(Si_4_O_10_)** |
| **PFl-1** | **Palygorskite** | | **Gadsden County, FL, USA** | **(Mg,Al)_2_Si_4_O_10_(OH)_4_H_2_O** |

**Table S3: Summary of chemical and physical characterizations of clay samples [30**]

| **Sample Designation** | **Clay mineral** | **Zeta potential (ZP), (mV)** | **γ_s_^-^** | **Water affinity** | **Interaction energy, (AB)** | **Interaction energy (vdW)** | **Interaction energy (Total)** | **Flocculation/ Dispersion in water** |
| --- | --- | --- | --- | --- | --- | --- | --- | --- |
| **SWy-3** | **Na-montmorillonite** | **-31.9** | **44.6** | **Hydrophilic / Polar** | **22400** | **-730** | **22800** | **Dispersion** |
| **PFl-1** | **Palygorskite** | **-24.2** | **23.2** | **Mildly Hydrophobic** | **-3100** | **-230** | **-2690** | **Flocculation** |

***Table S4: The effects of nano-clays on melanoma cell proliferation and viability – 3-day dosage r***esponse

| **Treatment** | **Mean Viable Cells** | **Mean Viability (%)** |
| --- | --- | --- |
| **Control** | 295200 | 100% |
| **2 μg/ml pFI-1** | 195200 | 68% |
| **5 μg/ml pFI-1** | 168000 | 52% |
| **10 μg/mlpFI-1** | 76800 | 26% |
| **2 μg/ml Swy-3** | 200800 | 66% |
| **5 μg/ml Swy-3** | 139200 | 58% |
| **10 µg/ml Swy-3** | 72800 | 21% |

*Notes.* Mean number of SK-Mel-28 melanoma cells and cell viability for treatments at concentrations of 0 (control), 2, 5, and 10μg/ml, respectively, three days post-treatment.

The data demonstrated significant differences in viable cell counts and viability:

10 μg/ml pFI-1 vs control, p < 0.0001

5 μg/ml pFI-1 vs control, p < 0.0001

2 μg/ml pFI-1 vs control, p < 0.0001

10 μg/ml Swy-3 vs control, p < 0.0001

5 μg/ml Swy-3 vs control, p < 0.0001

2 μg/ml Swy-3 vs control, p < 0.0001

**Table S5: *The effects of nano-clays on melanoma cell proliferation and viability – 5-day timing course***

|  | **Control** | | **10 μg/ml pFI-1** | | **10 μg/ml Swy-3** | | **10 μg/ml Mix** | |
| --- | --- | --- | --- | --- | --- | --- | --- | --- |
| **Days** | Viable Cells | Viability (%) | Viable Cells | Viability (%) | Viable Cells | Viability (%) | Viable Cells | Viability (%) |
| **1** | 114133 | 100% | 46933 | 35% | 32000 | 30% | 46333 | 32% |
| **3** | 497466 | 99% | 106400 | 30% | 88666 | 26% | 131250 | 31% |
| **5** | 888800 | 99% | 236866 | 37% | 188466 | 31% | 231916 | 31% |

*Notes.* Mean number of viable cells of SK-Mel-28 melanoma and cell viability for each treatment group, at 10 μg/ml, on days 1, 3, and 5 during a 5-day timing course.

There are significant differences in mean viable cell counts between the control group and treatment groups across all three days (all p <0.0001).

***Table S6: The effects of nano-clays treatments on tumor volume and w***eight

| **Treatment Regimen** | **Tumor Volume (mm^3^)** | | | **Tumor Weight (mg)** | | |
| --- | --- | --- | --- | --- | --- | --- |
|  | Mean | Std. Error | *P-value | Mean | Std. Error | *P value |
| **One-Time Treatment** | 39.31 | 7.22 | 0.0047 | 12.60 | 3.39 | 0.015 |
| **Weekly Treatment** | 38.36 | 9.30 | 0.0047 | 10.86 | 2.40 | 0.0085 |
| **Twice a Week Treatment** | 50.99 | 11.23 | 0.0093 | 15.04 | 3.66 | 0.0271 |
| **Daily Treatment** | 30.79 | 5.12 | 0.0012 | 15.27 | 2.04 | 0.0135 |
| **Control** | 182.02 | 40.35 |  | 38.14 | 8.27 |  |

*Notes.* Mean tumor weight and tumor volume after treatment one time on initial treatment day, weekly for four weeks, twice a week for four weeks, daily for three weeks, and no treatment (control), respectively.*P values were calculated by conducting a student’s t-test comparing the respective treatment group to the control. There were no significant differences amongst treatment groups.

***Table S7: The effects of nano-clays on melanoma cell d***ivision

| **Treatments** | **Mean Number of Cells Undergoing Mitosis** | **Std. Error** | ***P-Value** |
| --- | --- | --- | --- |
| **One-Time Treatment** | 2.80 | 0.73 | <0.0001 |
| **Weekly Treatment** | 0.00 | 0.00 | <0.0001 |
| **Twice a Week Treatment** | 1.60 | 0.58 | <0.0001 |
| **Daily Treatment** | 1.50 | 0.52 | <0.0001 |
| **Control (no treatment)** | 8.60 | 0.83 | <0.0001 |

*Notes.* Mean number of melanoma cells undergoing mitosis per field. Calculated by counting the number of cells undergoing mitosis in ten fields at 200 magnification.

The data analysis of differences in the mean number of cells undergoing mitosis:

Daily treatment vs control, p < 0.0001

Twice a week treatment vs control, p < 0.0001

Weekly treatment vs control, p < 0.0001

One-time treatment vs control, p < 0.0001

Weekly treatment vs daily treatment, p < 0.05

Weekly treatment vs twice a week treatment, p < 0.05

Weekly vs one-time treatment, p < 0.01

**Fig. S1**: SEM images and the corresponding molecular structure of the clays showing the configuration, isomorphous substitution, charge deficiency, and interlayer cations of different types of clays, (a) & (c) montmorillonite (drawn in Materials Studio software 2013), and (b) & (d) palygorskite (drawn in Materials Studio software 2013) (from [31])

**Fig. S2**: Particle size analysis of the clay samples from The Clay Minerals Society (CMS) in deionized water and RPMI using DLS technique after a series of processes including deflocculation, centrifugation, and sedimentation under gravity of the natural samples (from [31]).

**Fig. S3**: X-ray diffraction analysis of clay samples, comparing (a) natural samples obtained from the Clay Minerals Society, and (b) pure clay samples (from [31]).

**Fig. S4:** (a) Schematic of the AFM adhesive force measurements, showing (I) approach of the cantilever to the substrate, (II) establishment of contact, (III) retraction of the cantilever, and (IV) separation from the substrate. (b) Representative force traces, obtained for a cell-coated cantilever probe and the cells on the substrate (from [31]).


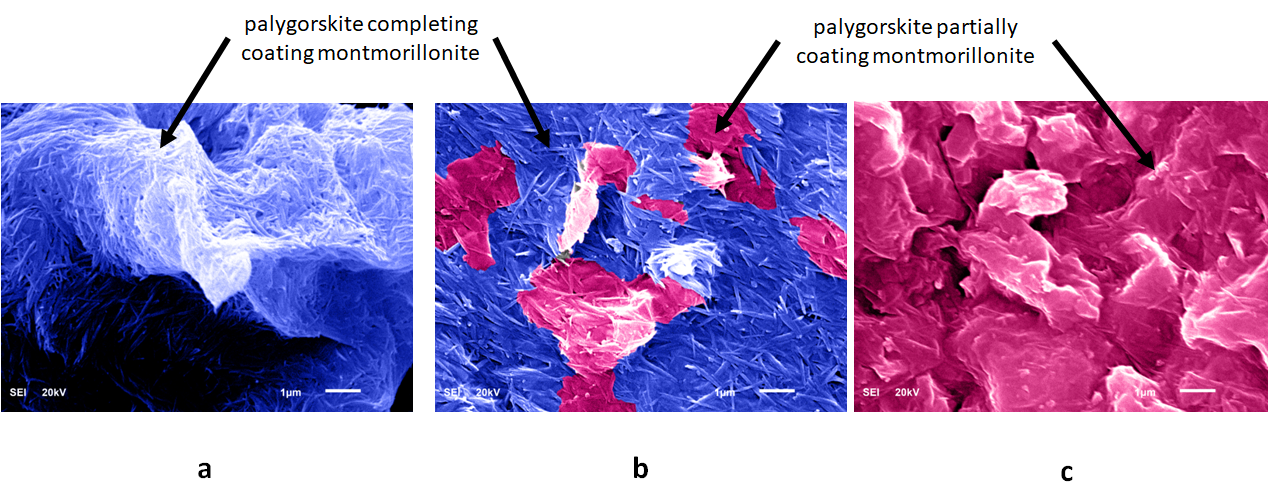


**Fig. S5**: SEMs of the Na-montmorillonite and palygorskite clay nanoparticles at different proportions (blue color depicts the predominance of palygorskite, while red color indicates the predominant Na-montmorillonite), (a) mont/play = 25/75: palygorskite particles could be seen completely covering the Na-montmorillonite particles, (b) mont/paly = 50/50: almost all the Na-montmorillonite particles are covered by palygorskite except some areas where the coating is lesser dense, (c) mont/paly = 75/25: palygorskite can be seen partially coating the Na-montmorillonite particles. (from [31])


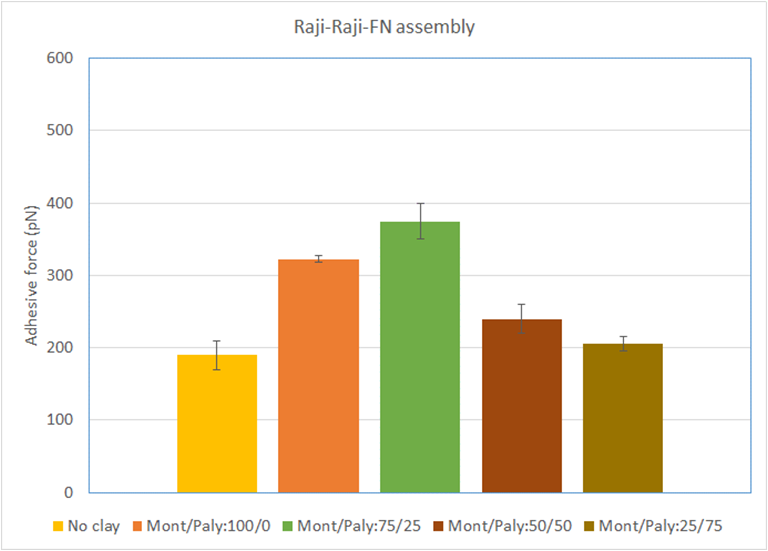


**Fig. S6**: Summary of adhesion force measurements among Raji-Raji-FN assembly using AFM, before and after treatment with various proportions of Na-montmorillonite and palygorskite clay nanoparticles. Error bars represent the variations in the trials. (from [31])
